# The Clinical, Radiological, and Laboratory Profile of Patients With Interstitial Pneumonitis With Autoimmune Features From India: An Observational, Cross-Sectional Study

**DOI:** 10.7759/cureus.15187

**Published:** 2021-05-23

**Authors:** Sheetal Chaurasia, Alamelu Haran, Pavny Chawla, Anish Reddy

**Affiliations:** 1 Pulmonary Medicine, Manipal Hospital Whitefield, Bangalore, IND; 2 Pulmonary Medicine, Vydehi Institute of Medical Sciences and Research Centre, Bangalore, IND; 3 Respiratory Diseases and Sleep Disorders, Artemis Hospital, Gurgaon, IND

**Keywords:** interstitial pneumonia with autoimmune features, interstitial lung disease, india, autoimmune, idiopathic interstitial pneumonia

## Abstract

Introduction

Interstitial pneumonia with autoimmune features (IPAF) refers to interstitial lung disease (ILD) with co-existing features of other clinical, serologic, or pulmonary features that suggest the presence of an underlying systemic autoimmune condition that does not fulfill the current rheumatologic criteria for connective tissue disorder (CTD). It is a relatively newly described clinical syndrome with only a handful of reports describing it. This study aimed at studying the clinical, radiological, and laboratory profiles of IPAF patients from a tertiary care hospital in South India.

Methods

This was an observational cross-sectional study conducted in a tertiary care hospital in South India over a period of one year. Patients diagnosed to have IPAF as per the European Respiratory Society (ERS)/American Thoracic Society (ATS) criteria were included in the study, and their demographics, clinical features, radiological features, and laboratory markers were collected along with a descriptive analysis.

Results

A total of 14,433 patients were screened during the study period. Twenty-four patients were diagnosed to have IPAF during the study period with a prevalence of 0.17%. Out of these 24 patients, 11 (45.8%) patients were males. The mean (M) ± standard deviation (SD) age was 47.8±10.7 years. Twenty-one (87.5%) of the patients reported having a cough, 18 (75%) patients had breathlessness, and 10 (41.7%) patients had digital clubbing. On radiological imaging, five (20.8%) patients had features of usual interstitial pneumonia (UIP) and 14 (58.3%) had nonspecific interstitial pneumonia (NSIP). On pulmonary function testing, the M±SD forced expiratory volume in the first second (FEV1) was 56.4±13.9%. The M±SD forced vital capacity (FVC) was 44.2±24.1%. The M±SD FEV1/FVC ratio was 0.8±0.04. On performing diffusing capacity of the lungs for carbon monoxide (DLCO), the M±SD was 34.2±21.9%. Of the patients, 95.8% had a positive antinuclear antibody (ANA) while 25% of patients had a positive anti-AMA-M2.

Conclusions

The prevalence of IPAF in the studied population was very low. IPAF had nonspecific clinical features, pulmonary function tests, and radiological findings. Further large-scale studies are required from different parts of the world in order to understand the epidemiology of IPAF. Research is also required into developing effective management options for IPAF.

## Introduction

In 2015, the European Respiratory Society (ERS)/American Thoracic Society (ATS) research statement coined a new term “interstitial pneumonia with autoimmune features” (IPAF) to describe interstitial lung disease (ILD) with co-existing features of other clinical, serologic, or pulmonary features that suggest the presence of an underlying systemic autoimmune condition that does not fulfill the current rheumatologic criteria for connective tissue disorder (CTD) [1]. They also introduced new classification criteria that would classify certain idiopathic ILDs into this new clinical entity.

Idiopathic interstitial pneumonias (IIPs) include diffuse inflammatory fibrotic lung disease, which is diagnosed based on having similar clinical, radiological as well as histopathologic features [2-4]. IIP is essentially a diagnosis of exclusion once other causes of interstitial pneumonia, including environmental exposure, drug toxicity, and CTD, are excluded as the underlying cause [3]. The ERS/ATS task force has now renamed these IIPs, which are likely of autoimmune etiology but are not part of CTD as IPAF. However, the clinical, radiological, and laboratory profile of patients with IPAF in India is poorly studied. This study aimed at characterizing these profiles in a cohort of IPAF patients from South India.

## Materials and methods

Design and setting

This was an observational, cross-sectional study that aimed at characterizing the clinical, radiological, and laboratory profiles of IPAF patients from India. This study was conducted in a tertiary care hospital in Bengaluru, South India, for a period of one year between December 2016 and December 2017. Any patient who visited the pulmonary medicine outpatients department of our hospital and fulfilled the ERS/ATS criteria for IPAF were included in the study. The nature of illness of the patients presenting to this outpatient clinic was varied and included new visits, re-visits, and referrals for a wide range of respiratory symptoms and disorders. Patients with an apparent underlying diagnosis that excluded IPAF were excluded from the study. Informed consent was obtained from every patient, and ethical clearance was obtained from the institutional ethical board prior to the initiation of the study. 

Definitions and methods

IPAF was diagnosed based on the ERS/ATF criteria, which are summarized in Table 1 [1].

**Table 1:** ERS/ATS criteria for the diagnosis of IPAF ERS: European Respiratory Society; ATS: American Thoracic Society; IPAF: interstitial pneumonia with autoimmune features; HRCT: high-resolution computed tomography; ANA: antinuclear antibody; NSIP: non-specific interstitial pneumonia; OP: organizing pneumonia; LIP: lymphoid interstitial pneumonia; PFT: pulmonary function testing

Presence of interstitial pneumonia (by HRCT or surgical lung biopsy), exclusion of alternative aetiologies, does not meet the criteria of a defined connective tissue disease, and at least one feature from at least two of these domains: Clinical domain, serologic domain, morphologic domain
Clinical domain: Distal digital fissuring (i.e. “mechanic hands”), distal digital tip ulceration, inflammatory arthritis or polyarticular morning joint stiffness ⩾60 min, palmar telangiectasia, Raynaud’s phenomenon, unexplained digital edema, unexplained fixed rash on the digital extensor surfaces (Gottron’s sign)
Serologic domain: ANA ⩾1:320 titer, diffuse, speckled, homogeneous patterns or ANA nucleolar pattern (any titer), or ANA centromere pattern (any titer), rheumatoid factor ⩾2× upper limit of normal, anti-CCP, anti-dsDNA, anti-Ro (SS-A), anti-La (SS-B), anti-ribonucleoprotein, anti-Smith, anti-topoisomerase (Scl-70), anti-tRNA synthetase (Jo-1, PL-7, PL-12, EJ, OJ, KS, Zo, tRS), anti-PM-Scl, anti-MDA-5
Morphologic domain: Suggestive radiology patterns by HRCT, including NSIP, OP, NSIP with OP overlap, LIP. Histopathology patterns or features by surgical lung biopsy of NSIP, OP, NSIP with OP overlap, LIP, interstitial lymphoid aggregates with germinal centers, diffuse lymphoplasmacytic infiltration (with or without lymphoid follicles). Multi-compartment involvement (in addition to interstitial pneumonia), including unexplained pleural effusion or thickening, unexplained pericardial effusion or thickening, unexplained intrinsic airways disease (by PFT, imaging, or pathology), unexplained pulmonary vasculopathy.

Once a patient fulfilled the criteria for IPAF, their demographics, clinical features, radiological features, and laboratory markers, including pulmonary function testing (PFT), diffusing capacity of the lungs for carbon monoxide (DLCO), six-minute walk test (6MWT), and serum autoimmune profile were collected into a Microsoft Excel 2016 (Microsoft Corporation, Albuquerque, New Mexico) by the lead investigator.

Statistical analysis

Statistical analysis was performed using Microsoft Excel 2016. Continuous variables were described by using mean ± standard deviation and proportions for categorical variables. Where appropriate, descriptive analysis was performed.

## Results

Demographics and clinical features

A total of 14,433 patients visited the pulmonary medicine outpatient department during the study period. Out of these, 24 patients were diagnosed to have IPAF as per the ERS/ATS criteria. The prevalence of IPAF was 0.17%. Out of these 24 patients, 11 (45.8%) patients were males while 13 (54.2%) patients were females. Ages ranged between 26 and 68 years with a mean (M) ± standard deviation (SD) of 47.8±10.7 years. Twenty-one (87.5%) patients reported having a cough at the time of presentation. Eighteen (75%) patients reported having varying degrees of breathlessness. Four (16.7%) patients reported having a low-grade, persistent fever, and one (4.2%) patient reported having pain in the small joints. Two (8.3%) patients had early morning stiffness. Three (12.5%) patients had digital fissuring. Ten (41.7%) patients had digital clubbing. Four (16.7%) patients had Raynaud's phenomenon. Eight (33.3%) patients had features suggestive of inflammatory arthritis at the time of presentation (Table 2).

**Table 2:** Demographic and clinical profile of the patients in the study ^1^ Expressed as mean ± standard deviation

PARAMETER	n (24)	Percentage (%)
Male gender	11	45.8
Age (years) ^1^	47.8±10.7	Not applicable
Cough	21	87.5
Breathlessness	18	75
Fever	4	16.7
Arthralgia	1	4.2
Early morning stiffness	2	8.3
Digital fissuring	3	12.5
Digital clubbing	10	41.7
Raynaud's phenomenon	4	16.7
Inflammatory arthritis	8	33.3

Radiological features

On high-resolution computed tomography (HRCT) thorax, five (20.8%) patients had features of usual interstitial pneumonia (UIP). Fourteen (58.3%) patients had features of nonspecific interstitial pneumonia (NSIP). Four (16.7%) patients had features of interstitial fibrosis. One (4.2%) patient had features suggestive of bronchiolitis obliterans organizing pneumonia (BOOP) (Table 3).

**Table 3:** Radiological features of the patients in the study

PARAMETER	n (24)	Percentage
Usual interstitial pneumonia	5	20.8
Nonspecific interstitial pneumonia	14	58.3
Interstitial fibrosis	4	16.7
Bronchiolitis obliterans organizing pneumonia	1	4.2

Laboratory features

On performing PFT, the forced expiratory volume in the first second (FEV1) ranged between 37% and 80% with an M±SD of 56.4±13.9%. The forced vital capacity (FVC) ranged between 24% and 77% with an M±SD of 44.2±24.1%. The FEV1/FVC ratio ranged between 0.7 and 0.9 with an M±SD of 0.8±0.04. On performing DLCO, the DLCO values ranged between 24% and 72% with an M±SD of 34.2±21.9%. On performing the 6MWT, the distance walked ranged between 90 and 310 meters with an M±SD of 181.8±85 meters. The CRP values ranged between 0.1% and 6.1 mg/dL with an M±SD of 2.1±1.5 mg/dL (Table 4). The frequency of the presence of the various antibodies in the autoimmune profile are summarized in Table 4.

**Table 4:** The laboratory parameters of the patients in the study 1 Expressed as mean ± standard deviation ANA: antinuclear antibody; RF: rheumatoid factor; CRP: C-reactive protein

PARAMETER	n (24)	Percentage
Forced expiratory volume in the first second (%) ^1^	56.4±13.9	Not applicable
Forced vital capacity (%) ^1^	44.2±24.1	Not applicable
Forced expiratory volume in the first second/Forced vital capacity ratio^1^	0.8±0.04	Not applicable
Diffusing capacity of the lungs for carbon monoxide (%) ^1^	34.2±21.9	Not applicable
Six-minute walk test distance (meters) ^1^	181.8±85	Not applicable
CRP (mg/dL)^1^	2.1±1.5	Not applicable
ANA	23	95.8
Anti-SSB	1	4.2
Anti-tRNA synthetase	2	8.3
Anti-AMA-M2	6	25
Anti-SSA	4	16.7
Anti-CENP-B	2	8.3
Anti-scl-70	3	12.5
Anti-PCNA	0	0
Anti-Jo-1	1	4.2
Anti-PM/Scl	1	4.2
Ati-Ro-52	1	4.2
RF	3	12.5
Anti-RNP	1	4.2
Anti-Sm	2	8.3
Anti-U1RNP	1	4.2

## Discussion

This was an observational, cross-sectional study conducted among outpatients visiting the pulmonary medicine department of a tertiary care center in South India. Patients who fulfilled the ERS/ATS criteria of IPAF were included in the study. The prevalence of IPAF in the present study from a section of patients who visited the pulmonary medicine outpatients department was found to be 0.17%. The prevalence of ILD was estimated to be 0.08% in the general population of the United States and 0.09 in France [5-6]. The prevalence of IPAF ranged between 7.3% and 34.1% of all ILDs in different reports [7-9]. However, the crude prevalence of IPAF has not been widely reported.

The gender distribution in our study was more or less equal but with a female predominance. The gender distribution in the other reports on IPAF too showed a female predominance with one study reporting 71% of the study participants to be women [8-9]. The mean age was slightly higher at 63 years compared to our 48 years in a study from the United States (US) [9].

The predominant symptom in our cohort included cough and breathlessness in a large majority of the patients with some patients reporting low-grade fever, arthralgia, and early morning stiffness. Digital clubbing, fissuring, Raynaud's phenomenon, and small joint inflammatory arthritis were also noted at varying proportions in our patients. A study from the US reported Raynaud's phenomenon in 39% of cases, digital fissuring in 29%, Gottron's sign in 18%, and inflammatory arthritis in 16% of patients [8]. Our findings resembled the findings of this study. A study from the US reported digital clubbing about 20% of their IPAF patients compared to the 41% found in our study indicating that clubbing may be a useful clinical sign in IPAF [9].

Radiologically, our study found the predominant HRCT finding to be suggestive of NSIP in about 59% of cases followed by UIP in about 21% of cases. Similarly, the French study reported 53% of the IPAF patients to have NSIP [7]. A US study reported 57% NSIP in their cohort while the other US study reported only 32% NSIP in their cohort [8-9]. This suggests that NSIP may be the predominant radiological finding in IPAF.

The mean FEV1 and FVC in our study were reduced, at approximately 56% and 44% but with a normally maintained mean ratio of 0.8. The French study reported a mean FVC of 64% while the US study showed a mean FVC of 62% [7,9]. The mean DLCO in our study was around 34% while in other reports were 49% and 45% [7,9]. We also found gross limitations on the 6MWT, which was not reported in the previous studies.

Looking at the autoimmune profile, we found that almost all our patients had a positive ANA. One US study had a positive ANA in 77% of their patients while the other one had a positive ANA in approximately half their cohort. This heterogeneity may be due to the fact that ANA positivity is inherently higher in the Indian population as compared to the population of the US with previous studies reporting 33% positivity in the normal population in India as compared to around 14% in the US [10,11]. In our study, the anti-AMA-M2 antibody was found in 25% of patients while the anti-SSA antibody was found in 16.7% and a small proportion of patients had the other antibodies we tested for. A study from the US found anti-Ro (SSA) antibodies in 43% of their patients and anti-tRNA-synthetase antibodies in 36% of patients [8]. Another study from the US found anti-Ro (SSA) antibodies in about 17% of patients, with that being the second-highest reported antibody after ANA [9]. ANA and anti-Ro (SSA) antibodies may be useful in the diagnosis of IPAF.

Our study had limitations. Even though the study period was for a year, due to the extremely low prevalence of IPAF, we could identify only 24 patients during the study period. Studies with a longer duration are warranted to understand the distribution of the study parameters in a larger sample group. This was a single-center study and hence the prevalence calculated may not be accurate. We also sampled only outpatients reporting to pulmonary medicine, which could have led to a selection bias, as IPAF patients are more likely to present as compared to the general population.

## Conclusions

The prevalence of IPAF was small in the present study but more multi-centric studies are warranted to corroborate these findings. The clinical features, pulmonary function tests, and radiological findings in IPAF patients were nonspecific. The autoimmune markers could be a useful tool in the diagnosis of this condition in the background of typical clinical and radiological features. When IPAF is suspected, other autoimmune features should be actively searched for and addressed in order to improve the patient's quality of life. Since it is a relatively newly described syndrome, further research is required to assess the epidemiology as well as management options.

## References

[1] Fischer A, Antoniou KM, Brown KK (2015). An official European Respiratory Society/American Thoracic Society research statement: interstitial pneumonia with autoimmune features. Eur Respir J.

[2] American Thoracic Society, European Respiratory Society (2002). American Thoracic Society/European Respiratory Society International Multidisciplinary Consensus Classification of the idiopathic interstitial pneumonias. Am J Respir Crit Care Med.

[3] Raghu G, Collard HR, Egan JJ (2011). An official ATS/ERS/JRS/ALAT statement: idiopathic pulmonary fibrosis: evidence-based guidelines for diagnosis and management. Am J Respir Crit Care Med.

[4] Travis WD, Costabel U, Hansell DM (2013). An official American Thoracic Society/European Respiratory Society statement: update of the international multidisciplinary classification of the idiopathic interstitial pneumonias. Am J Respir Crit Care Med.

[5] Coultas DB, Zumwalt RE, Black WC, Sobonya RE (1994). The epidemiology of interstitial lung diseases. Am J Respir Crit Care Med.

[6] Duchemann B, Annesi-Maesano I, Jacobe de Naurois C (2017). Prevalence and incidence of interstitial lung diseases in a multi-ethnic county of Greater Paris. Eur Respir J.

[7] Ahmad K, Barba T, Gamondes D (2017). Interstitial pneumonia with autoimmune features: clinical, radiologic, and histological characteristics and outcome in a series of 57 patients. Respir Med.

[8] Chartrand S, Swigris JJ, Stanchev L, Lee JS, Brown KK, Fischer A (2016). Clinical features and natural history of interstitial pneumonia with autoimmune features: a single center experience. Respir Med.

[9] Oldham JM, Adegunsoye A, Valenzi E (2016). Characterisation of patients with interstitial pneumonia with autoimmune features. Eur Respir J.

[10] Gupta P, Priya R, Nanda R, Patel S, Mohapatra E (2020). A hospital-based insight into the antinuclear antibody patterns in autoimmune disorders. J Lab Physicians.

[11] Satoh M, Chan EK, Ho LA (2012). Prevalence and sociodemographic correlates of antinuclear antibodies in the United States. Arthritis Rheum.

